# Association of sex with post-arrest care and outcomes after out-of-hospital cardiac arrest of initial shockable rhythm: a nationwide cohort study

**DOI:** 10.3389/fcvm.2023.1269199

**Published:** 2024-01-04

**Authors:** Sanae Hosomi, Taro Irisawa, Shunichiro Nakao, Ling Zha, Kousuke Kiyohara, Tetsuhisa Kitamura, Hiroshi Ogura, Jun Oda

**Affiliations:** ^1^ Department of Traumatology and Acute Critical Medicine, Osaka University Graduate School of Medicine, Osaka, Japan; ^2^ Division of Environmental Medicine and Population Sciences, Department of Social Medicine, Graduate School of Medicine, Osaka University, Osaka, Japan; ^3^ Department of Food Science, Faculty of Home Economics, Otsuma Women’s University, Tokyo, Japan

**Keywords:** sex, shockable rhythm, out-of-hospital cardiac arrest, in-hospital treatment, survival outcome

## Abstract

**Background:**

Research has described differences in the provision of prehospital treatment for women who experience out-of-hospital cardiac arrest. However, studies have reported conflicting results regarding survival outcomes or in-hospital interventions between sexes. Thus, this study aimed to investigate the association of sex with survival outcomes and in-hospital treatments in Japan.

**Methods:**

We retrospectively analyzed data from the Japanese Association for Acute Medicine–Out-of-Hospital Cardiac Arrest Registry. Patients aged ≥18 years who presented with a shockable rhythm at the scene between June 2014 and December 2020 were included in our analysis. Outcome measures were 30-day survival and in-hospital interventions. We compared the outcomes between the sexes using multivariable logistic regression.

**Results:**

In total, 5,926 patients (4,270 men; 1,026 women) with out-of-hospital cardiac arrest were eligible for our analysis. The proportions of patients with 30-day survival outcomes were 39.5% (1685/4,270) and 37.4% (384/1,026) in the male and female groups, respectively (crude odds ratio, 0.92; 95% confidence interval, 0.80–1.06). Although there were no significant differences, survival outcomes tended to be better in women than in men in the multiple regression analysis (adjusted odds ratio: 1.38; 95% confidence interval: 0.82–2.33). Furthermore, there was no significant difference between the sexes in terms of patients who received extracorporeal cardiopulmonary resuscitation (adjusted odds ratio: 0.81; 95% confidence interval: 0.49–1.33) or targeted temperature management (adjusted odds ratio: 0.99; 95% confidence interval: 0.68–1.46).

**Conclusions:**

After adjusting for prognostic factors, there were no differences in survival rates and in-hospital interventions between men and women.

## 1 Introduction

Out-of-hospital cardiac arrest (OHCA) is a major public health concern worldwide with high occurrence and mortality rates (1–3). In Japan, there are approximately 70,000 cases of OHCA annually (4, 5). The incidence of emergency medical services (EMS)-treated OHCA in the United States is estimated to be 356,461, with nearly 90% of the cases being fatal (6). The overall prognosis and neurological outcomes are relatively poor following OHCA, and survival to hospital discharge after EMS-treated cardiac arrest is approximately 10% (1–6). The American Heart Association-International Liaison Committee on Resuscitation advocates the “chain of survival” model, which emphasizes the need for timely access to medical care and early intervention (7). Moreover, the guidelines address sex-related inequalities, with female victims less likely to receive bystander cardiopulmonary resuscitation (CPR), which is a major issue (7).

In recent years, there has been growing interest in examining the differences in the prognosis of various diseases between men and women (8–13). Research has found sex-related disparities in pathophysiology, clinical symptoms, and outcomes, as well as in medical care received (8–13). Cardiovascular disease is the most extensively analyzed topic of sex-related differences. Studies have shown that there is a variation in clinical outcomes after percutaneous coronary intervention (PCI) for acute myocardial infarction between sexes (14). Women appear to have a greater risk of heart failure following PCI for acute myocardial infarction than men, despite having the same technical success rate (15). Studies also suggest that women are less likely to receive mechanical cardiac support or undergo diagnostic procedures such as coronary angiography or PCI (16). Researchers have conducted multiple studies on possible sex differences associated with OHCA prognosis; however, their findings have been inconsistent (17, 18). Whether sex-based variations affect the prognosis of OHCA is unclear.

The treatment of OHCA in hospitals has seen a surge in specialized interventions, such as extracorporeal cardiopulmonary resuscitation (ECPR), targeted temperature management (TTM), and PCI, making them standard protocols globally (19, 20). Despite these advances, research on sex-related differences in in-hospital treatment and post-resuscitation care is lacking. Studies conducted in Japan indicate that women aged 18–64 years are less likely to receive CPR in public, and men are more likely to receive aggressive prehospital treatment (21, 22). Therefore, we hypothesized that women in Japan receive fewer in-hospital treatments or have poorer survival outcomes than men. This study aimed to explore sex-associated differences in in-hospital treatment and survival outcomes in Japan using the JAAM-OHCA registry, a multicenter prospective database.

## 2 Methods

### 2.1 Study design and setting

We retrospectively analyzed data from the JAAM-OHCA registry, a Japanese multicenter nationwide prospective database that includes prehospital and in-hospital information and outcomes of patients with OHCA transported to the emergency departments of the participating institutions. The ongoing registry was started in June 2014 and currently has no anticipated end date. It includes 95 institutions: 71 university hospitals and/or critical care medical centers and 24 community hospitals providing emergency care (23). The registry includes all patients with OHCA who required resuscitation by EMS and were transported to the participating institutions. The detailed methodology of the JAAM-OHCA registry has been described previously (24). Briefly, EMS collected prehospital data according to the international Utstein style (25), in-hospital data were collected by the medical staff of each institution in accordance with a standardized format using an Internet-based system, and the prehospital and in-hospital information was integrated by the JAAM-OHCA registry committee. The causes of arrest were classified as cardiac or noncardiac. The presumed cardiac cause category was determined by exclusion (i.e., the diagnosis was made when there was no evidence of a noncardiac cause) based on the Utstein style guidelines. Cardiac or noncardiac origin was clinically determined by the physician in charge.

### 2.2 Patient selection

This study enrolled the following patients: those aged ≥18 years who sustained cardiac arrest in a prehospital setting, patients for whom resuscitation was attempted, and those who were then transported to the participating institutions in Japan from 1 June 2014 to 31 December 2020. Cases of noncardiac origin were excluded because their outcomes differed (4). Cases of non-shockable rhythm at the scene were also excluded because shockable rhythms provide a suitable comparator for judging the success of systems nationally and internationally, as previous adult Utstein templates focused on witnessed ventricular fibrillation (VF) arrests (25). The ethics committee of each participating institution approved the study protocol.

### 2.3 EMS organization in Japan

The details of the EMS system in Japan have been previously described (2–4). In brief, the EMS system in Japan is managed by local fire stations and provides emergency services 24/7. Emergency life-saving technicians (ELSTs) are highly trained prehospital emergency care personnel who work in teams of three professionals per ambulance. ELSTs are authorized to perform various medical procedures, including the use of an intravenous line, an advanced airway, and a semi-automated external defibrillator (AED). Specially trained ELSTs are also permitted to perform tracheal intubation and administer intravenous adrenaline. CPR is performed according to the Japanese CPR guidelines, and living wills or do-not-resuscitate orders are not widely accepted. EMS personnel are not authorized to terminate resuscitation in the field, and patients with OHCA without rigor mortis, incineration, decomposition, decapitation, or dependent cyanosis are transported to the hospital for further treatment.

### 2.4 Data collection and outcome measures

The following data were obtained from the JAAM-OHCA registry: sex, age, cause of arrest, arrest witnessed by bystanders, bystander-initiated CPR, first documented rhythm, resuscitation time course, actual treatments in prehospital and hospital settings (e.g., adrenaline, TTM, PCI, and ECPR), and outcome data. In cases of shock delivery by bystanders using a public-access AED, the patient's first recorded rhythm was considered pulseless ventricular tachycardia (VT) or VF. The outcome measure was 1-month survival or 1-month survival with favorable neurological outcomes. Neurological outcomes were evaluated using the cerebral performance category (CPC) scale. A favorable neurological outcome was defined as CPC 1 or 2 (2–4). Furthermore, sex disparities related to in-hospital interventions (adrenaline, antiarrhythmic drugs (amiodarone, lidocaine, nifekalant, magnesium), coronary artery angiography [CAG], PCI, intra-aortic balloon pump [IABP], ECPR, and TTM) were analyzed.

### 2.5 Statistical analysis

Categorical variables are presented as counts with proportions, and the *χ*^2^ test was used to evaluate differences between the two groups. Continuous variables are presented as medians with interquartile ranges, and the Mann–Whitney *U*-test was used to evaluate differences between the two groups.

Furthermore, multiple logistic regression analysis was used to assess factors associated with survival outcomes or in-hospital interventions, and adjusted odds ratios (AORs) and 95% confidence intervals (CIs) were calculated. As potential confounders, factors that were biologically essential and considered to be associated with clinical outcomes were included in the multivariate analyses (2–4, 24, 26, 27). The variables included age (grouped as 18–19, 20–29, 30–39, 40–49, 50–59, 60–69, 70–79, 80–89, and ≥90 years), witness status (yes/no), origin (coronary disease/others/unknown), daytime (9:00 am–4:59 pm) (yes/no), weekend/holiday (yes/no), use of an AED (yes/no), bystander chest compression (yes/no), advanced airway management by EMS (yes/no), administration of adrenaline by EMS (yes/no), return of spontaneous circulation (ROSC) status (after hospital arrival, at hospital arrival, no ROSC), in-hospital first documented rhythm [VF or pulseless VT/pulseless electrical activity (PEA) or asystole/presence of pulse], time from call to hospital arrival, and year of onset. For another model for survival outcome analysis, additional variables such as antiarrhythmic drugs (yes/no), CAG (yes/no), IABP (yes/no), ECPR (yes/no), and TTM (yes/no) were added. A subgroup analysis of in-hospital treatments was also performed in terms of in-hospital treatments by narrowing based on treatment received (in particular, patients with ST-elevation on 12-lead electrocardiogram [ECG] after ROSC received CAG or PCI; VF/pulseless VT patients with first documented rhythm at hospital arrival received ECPR, adrenaline, or antiarrhythmic drugs; and patients with ROSC after/at hospital arrival received TTM or IABP).

All statistical analyses were performed using STATA version 16 (StataCorp LP, College Station, TX, USA). All tests were two tailed, and *p*-values <0.05 were considered statistically significant.

### 2.6 Ethics approval

This manuscript complies with the STROBE statement for the reporting of cohort and cross-sectional studies (28). The study design was approved by the Ethics Committee of the Osaka University Graduate School of Medicine (approval number: 21304-3). The requirement for written informed consent was waived due to the retrospective nature of the study. Personal identifiers were not included in the JAAM-OHCA records.

## 3 Results

**Figure 1:**
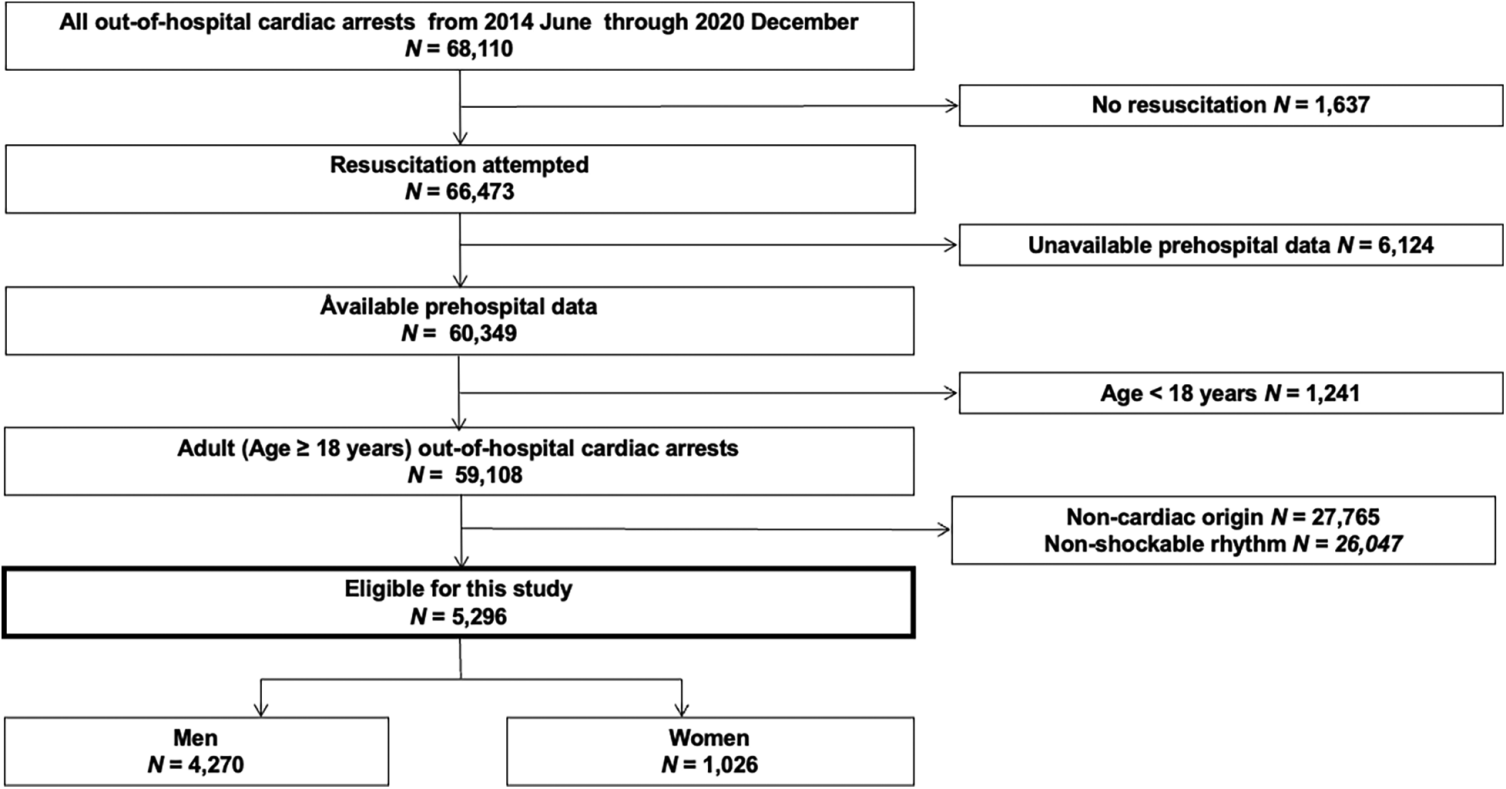
Flow chart of the patient inclusion process used in this study.

A flow chart of the patient selection process is shown in Figure 1. During the study period, 68,110 OHCA cases were documented in the JAAM-OHCA registry. Among them, 5,296 adult patients (4,270 men and 1,026 women) with shockable rhythms at the scene were eligible for analysis.

Table 1 shows patient characteristics according to sex. Women were older and less likely to have coronary disease than men. A higher proportion of OHCA cases was observed in men than in women. The proportion of women who received shock from both a public AED and bystander-initiated chest compression or had an ROSC status was similar to that of men. Men were more likely to receive prehospital adrenaline administration and sustain VF/pulseless VT rhythm at hospital arrival than women. The time from call to hospital arrival was longer in men than in women.

**Table 1:** Characteristics of adults with out-of-hospital cardiac arrests of initial shockable rhythm in Japan.

		Total	Men	Women	*p*-Value
		*N* = 5,296	*N* = 4,270	*N* = 1,026	
Age, median (IQR), years	Median (IQR)	65 (53–75)	64 (53–73)	72 (56–82)	<0.001
Age group, *n* (%), years	18–64	2,515 (47.5%)	2,163 (50.7%)	352 (34.3%)	<0.001
	65–74	1,389 (26.2%)	1,174 (27.5%)	215 (21.0%)	
	75-	1,392 (26.3%)	933 (21.9%)	459 (44.7%)	
Witnessed, *n* (%)		3,997 (75.5%)	3,250 (76.1%)	747 (72.8%)	0.027
Cause, *n* (%)	Coronary Disease	1,979 (37.4%)	1,716 (40.2%)	263 (25.6%)	<0.001
	Others	1,475 (27.9%)	1,118 (26.2%)	357 (34.8%)	
	Unknown	1,842 (34.8%)	1,436 (33.6%)	406 (39.6%)	
Weekend, *n* (%)		1,785 (33.7%)	1,440 (33.7%)	345 (33.6%)	0.95
Daytime, *n* (%)		2,419 (45.7%)	1,960 (45.9%)	459 (44.7%)	0.50
Shock by a public-access AED, *n* (%)		1,017 (19.2%)	818 (19.2%)	199 (19.4%)	0.86
Bystander-initiated chest compression, *n* (%)		3,055 (57.7%)	2,463 (57.7%)	592 (57.7%)	0.99
Prehospital advanced airway management, *n* (%)		2,417 (45.6%)	1,958 (45.9%)	459 (44.7%)	0.52
Prehospital Adrenaline administration, *n* (%)		1,685 (31.8%)	1,387 (32.5%)	298 (29.0%)	0.034
Call to hospital arrival, median (IQR), min		37 (29–48)	37 (30–49)	36 (27–45)	0.018
Prehospital ROSC, *n* (%)		1,865 (35.2%)	1,492 (34.9%)	373 (36.4%)	0.39
ROSC status, *n* (%)	ROSC after hospital arrival	1,921 (36.3%)	1,578 (37.0%)	343 (33.4%)	0.11
	ROSC at hospital arrival	1,586 (29.9%)	1,263 (29.6%)	323 (31.5%)	
	No ROSC	1,789 (33.8%)	1,429 (33.5%)	360 (35.1%)	
First documented rhythm at hospital arrival, *n* (%)	VF/pulseless VT	1,625 (30.7%)	1,360 (31.9%)	265 (25.8%)	<0.001
	PEA/Asystole	2,183 (41.2%)	1,729 (40.5%)	454 (44.2%)	
	Presence of pulse	1,488 (28.1%)	1,181 (27.7%)	307 (29.9%)	

AED, automated external defibrillator; IQR, interquartile range; PEA, pulseless electrical activity; ROSC, return of spontaneous circulation; VF, ventricular fibrillation; VT, ventricular tachycardia.

Sex disparities in outcomes are shown in Table 2. Overall, 37.4% of women (384 of 1,026) and 39.5% of men (1,685 of 4,270) had 1-month survival (crude odds ratio [OR], 0.92; 95% CI: 0.80–1.06). In the multivariate logistic regression analyses, the outcome tended to be better in women than in men (AOR: 1.30; 95% CI: 0.81–2.09 after adjusting for only prehospital factors) (AOR: 1.38; 95% CI: 0.82–2.33 after adjusting for both prehospital and in-hospital factors), although it was not significantly different. In cases of 1-month survival with favorable neurological outcomes, the results were similar.

**Table 2:** Survival outcomes among adults with out-of-hospital cardiac arrests of initial shockable rhythm in Japan.

	Total	Men	Women
	*N* = 5,296	*N* = 4,270	*N* = 1,026
Admitted to ICU/ward, *n* (%)	3,525 (66.6%)	2,883 (67.5%)	642 (62.6%)
	Crude OR	Reference	0.80 (0.70–0.93)
	Adjusted OR (model 1)^a^	Reference	0.96 (0.53–1.74)
	Adjusted OR (model 2)^b^	Reference	0.67 (0.30–1.54)
1-month survival, *n* (%)	2,070 (39.1%)	1,686 (39.5%)	384 (37.4%)
	Crude OR	Reference	0.92 (0.80–1.06)
	Adjusted OR (model 1)^a^	Reference	1.30 (0.81–2.09)
	Adjusted OR (model 2)^b^	Reference	1.38 (0.82–2.33)
CPC 1 or 2 at 1 month after OHCA	1,507 (28.5%)	1,229 (28.8%)	278 (27.1%)
	Crude OR	Reference	0.92 (0.79–1.07)
	Adjusted OR (model 1)^a^	Reference	1.52 (0.90–2.57)
	Adjusted OR (model 2)^b^	Reference	1.65 (0.95–2.85)

AED, automated external defibrillator; CAG, coronary artery angiography; CI, confidence interval; CPC, cerebral performance category; ECPR, extracorporeal cardiopulmonary resuscitation; EMS, emergency medical services; IABP, intra-aortic balloon pump; ICU, intensive care unit; OHCA, out-of-hospital cardiac arrest; OR, odds ratio; PEA, pulseless electrical activity; ROC, receiver operating characteristic; ROSC, return of spontaneous circulation; TTM, targeted temperature management; VF, ventricular fibrillation; VT, ventricular tachycardia.

^a^ Model 1 included age (grouped into 10-year intervals), witness status, origin (coronary disease/others/unknown), daytime (9:00 am–4:59 pm) (yes/no), weekend/holiday (yes/no), use of an AED (yes/no), bystander chest compression (yes/no), advanced airway management by EMS (yes/no), adrenaline by EMS (yes/no), ROSC status (after hospital arrival, at hospital arrival, no ROSC), in-hospital first documented rhythm (VF or pulseless VT/PEA or asystole/presence of pulse), call to hospital arrival and year of onset. The area under the receiver operating characteristic (ROC) curve was 0.9234.

^b^ Model 2 included age (grouped into 10-year intervals), witness status, origin (coronary disease/others/unknown), daytime (9:00 am–4:59 pm) (yes/no), weekend/holiday (yes/no), use of an AED (yes/no), bystander chest compression (yes/no), advanced airway management by EMS (yes/no), adrenaline by EMS (yes/no), ROSC status (after hospital arrival, at hospital arrival, no ROSC), in-hospital first documented rhythm (VF or pulseless VT/PEA or asystole/presence of pulse), antiarrhythmic drug (yes/no), CAG (yes/no), IABP (yes/no), ECPR (yes/no), TTM (yes/no), call to hospital arrival and year of onset. The area under the ROC curve = 0.9395.

Sex disparities in in-hospital treatment are shown in Table 3. With a focus on highly specialized interventions, 13.0% of women (133 of 1,026) and 22.0% of men (939 of 4,270) received ECPR (crude OR: 0.53; 95% CI: 0.43–0.64). In contrast, there was no significant difference between the sexes in terms of those who received ECPR (AOR, 0.81; 95% CI, 0.49–1.33) in the multivariate logistic regression analyses. Similarly, the number of male patients who received TTM was 1,533 (35.9%), whereas the number of female patients who received TTM was 302 (29.4%) (crude OR: 0.74; 95% CI: 0.64–0.86; AOR: 0.99; 95% CI: 0.68–1.46).

**Table 3:** In-hospital treatments among adults with out-of-hospital cardiac arrests of initial shockable rhythm in Japan.

	Total	Men	Women
	*N* = 5,296	*N* = 4,270	*N* = 1,026
Adrenaline	3,473 (65.6%)	2,818 (66.0%)	655 (63.8%)
	Crude OR	Reference	0.91 (0.79–1.05)
	Adjusted OR	Reference	1.17 (0.69–1.98)
Antiarrhythmic drug^a^	1,791 (33.8%)	1,502 (35.2%)	289 (28.2%)
	Crude OR	Reference	0.72 (0.62–0.84)
	Adjusted OR	Reference	1.11 (0.75–1.66)
Coronary angiography	2,727 (51.5%)	2,288 (53.6%)	439 (42.8%)
	Crude OR	Reference	0.65 (0.56–0.74)
	Adjusted OR	Reference	1.04 (0.69–1.57)
Percutaneous coronary intervention	1,419 (26.8%)	1,255 (29.4%)	164 (16.0%)
	Crude OR	Reference	0.46 (0.38–0.55)
	Adjusted OR	Reference	0.89 (0.52–1.50)
Intra-aortic balloon pumping	1,344 (25.4%)	1,151 (27.0%)	193 (18.8%)
	Crude OR	Reference	0.63 (0.53–0.74)
	Adjusted OR	Reference	1.33 (0.88–2.02)
Extracorporeal Cardiopulmonary Resuscitation	1,072 (20.2%)	939 (22.0%)	133 (13.0%)
	Crude OR	Reference	0.53 (0.43–0.64)
	Adjusted OR	Reference	0.81 (0.49–1.33)
Targeted temperature management	1,835 (34.6%)	1,533 (35.9%)	302 (29.4%)
	Crude OR	Reference	0.74 (0.64–0.86)
	Adjusted OR	Reference	0.99 (0.68–1.46)

CI, confidence interval; OR, odds ratio.

^a^ Amiodarone, lidocaine, nifekalant, magnesium.

In the subgroup analysis focused on ST-segment elevation in 12-lead ECG after ROSC, in cases with VF/pulseless VT as the first documented rhythm at hospital arrival and in ROSC after/at hospital arrival, women were almost equally likely to receive in-hospital treatment when compared with men (Table 4).

**Table 4:** Subgroup analysis.

	Total	Men	Women
	*N* = 5,296	*N* = 4,270	*N* = 1,026
ST-elevation (12-lead ECG after ROSC)			
Coronary angiography	1,220 (82.9%)	1,051 (83.6%)	169 (78.6%)
	Crude OR	Reference	0.72 (0.50–1.03)
	Adjusted OR	Reference	1.56 (0.52–4.69)
Percutaneous coronary intervention	880 (59.8%)	776 (61.7%)	104 (48.4%)
	Crude OR	Reference	0.58 (0.43–0.78)
	Adjusted OR	Reference	0.88 (0.35–2.21)
VF/pulseless VT (First documented rhythm at hospital arrival)			
Extracorporeal Cardiopulmonary Resuscitation	690 (42.5%)	604 (44.4%)	86 (32.5%)
	Crude OR	Reference	0.60 (0.46–0.79)
	Adjusted OR	Reference	1.05 (0.57–1.97)
Adrenaline	1,338 (82.3%)	1,123 (82.6%)	215 (81.1%)
	Crude OR	Reference	0.91 (0.65–1.27)
	Adjusted OR	Reference	1.03 (0.46–2.30)
Antiarrhythmic drug^a^	1,052 (64.7%)	883 (64.9%)	169 (63.8%)
	Crude OR	Reference	0.95 (0.72–1.25)
	Adjusted OR	Reference	1.56 (0.85–2.87)
ROSC after hospital arrival/ROSC at hospital arrival			
Targeted temperature management	1,715 (48.9%)	1,432 (50.4%)	283 (42.5%)
	Crude OR	Reference	0.73 (0.61–0.86)
	Adjusted OR	Reference	0.88 (0.58–1.33)
Intra-aortic balloon pumping	1,180 (33.6%)	1,010 (35.6%)	170 (25.5%)
	Crude OR	Reference	0.62 (0.51–0.75)
	Adjusted OR	Reference	1.25 (0.78–2.01)

CI, confidence interval; ECG, electrocardiography; OR, odds ratio; ROSC, return of spontaneous circulation; VF, ventricular fibrillation; VT, ventricular tachycardia.

^a^ Amiodarone, lidocaine, nifekalant, magnesium.

## 4 Discussion

In this study, we evaluated the association between sex and survival outcomes of patients with OHCA with shockable rhythm at the scene or during in-hospital interventions using the JAAM-OHCA nationwide registry. There were no significant differences between women and men in terms of survival outcomes or hospital interventions, and this result was consistent in the subgroup analysis.

Several studies have examined the potential effect of sex on outcomes associated with OHCA and have reported varying results (17, 18, 29, 30). Some studies found no disparity between male and female survival rates following OHCA, whereas others found a survival advantage in males over females (30). According to a recent meta-analysis (31), women continue to exhibit substantially lower discharge survival rates and poorer neurological prognoses than men. Our findings, however, did not align with this conclusion, showing no statistically significant difference in survival outcomes between sexes. As noted in the meta-analysis, differences in medical care received after admission may play a role in the observed differences between male and female survival rates and should be considered when interpreting results from individual studies that do not account for this in-hospital factor adjustment. Furthermore, previous research conducted on sex-related differences in OHCA has shown significant variance in terms of the study participants' baseline characteristics and design, as a considerable proportion of patients with OHCA present with non-VF rhythm (31). This heterogeneity is a major bias in determining the sex-related differences described above. Hence, the outcomes of studies on sex-related disparity may not be as credible when combined or generalized because of selection and information bias. To simplify the diverse study design and baseline data, our study concentrated on sex differences in patients with OHCA with a shockable rhythm at the scene.

Our study showed that women tended to have better survival rates after adjusting for cardiac arrest. This OR reversal was also found in previous OHCA studies (32–34). Various factors, such as age, witnessed arrest, and bystander CPR, are associated with favorable outcomes in male patients with OHCA. Men also commonly experience an arrest of cardiac etiology and shockable rhythm as their initial cardiac rhythm, whereas women are more likely to have noncardiac etiologies (31). Consistent with this, the crude OR for survival outcome was also lower in women than in men in our study. However, after adjusting for prognostic factors, this analysis identified that survival outcomes tended to be better in women than in men. Therefore, we speculate that prehospital baseline factors and in-hospital care significantly affect OHCA survival rates between sexes, and previous studies may not have considered these factors, resulting in inconsistent findings. Contrary to our findings, a previous study conducted in the United States between 2003 and 2012 demonstrated that women had a higher risk of adjusted in-hospital mortality than men, particularly when diagnosed with VT/VF arrests (18). This could be due to the absence of prehospital information, interventions, or early in-hospital care in their research.

Reports have indicated varying rates of prevalence in channelopathies among different sexes (35). For instance, congenital long-QT syndrome has a higher predilection for women compared to men, putting women at a greater risk for Torsades de pointes and sudden cardiac death (36). Conversely, Brugada syndrome primarily affects adult men, who face a significantly elevated risk of arrhythmic, sudden cardiac death compared to women (37). This aspect holds significance for cardiomyopathies as well (38). Additionally, disparities in clinical characteristics may stem from societal and environmental factors that disadvantage women. According to previous reports, men tend to receive more post-admission interventions such as PCI, CAG, and TTM than women (39–41). Factors such as education level, religious beliefs, and economic level also contribute to women being more inclined to issue “do-not-resuscitate” instructions during OHCA, opting for more conservative treatment (42). One reason for this sex-related disparity might be that these treatments are invasive or expensive (43). Post-resuscitation care may also vary based on factors such as families' requests and professional concerns (17, 44). In contrast, our findings showed no significant difference in in-hospital treatment between the sexes. Furthermore, Japan's insurance system ensures that early cessation of expensive care for socioeconomic reasons is somewhat lower than that in other countries (45).

In our study, we showed that patients with OHCA with shockable rhythm who should be provided with highly specialized interventions have no sex-related disparity in survival outcomes or in-hospital treatment. Most previous studies have adjusted only for prehospital factors in patients with OHCA; thus, studies adjusted for in-hospital treatments such as care for post-cardiac arrest status are limited (31). Therefore, these results are considered appropriate. Our findings provide insights into sex-related disparities in patients with OHCA.

This study had some limitations. First, the Utstein style registry does not contain information on medications, medical history, and daily activities of each patient before cardiac arrest, which could affect the decision to pursue aggressive treatment options. Second, the registry does not provide detailed information on factors such as cardiovascular risk, symptom onset, and time to cardiac arrest, which might have skewed the results. Other potential factors, such as education level and sex inequality, may also play a role. Another limitation is the applicability of the study; as it is a single-country study, it would be intriguing to observe whether these findings hold true in a multinational study involving diverse patient populations and healthcare systems. Further investigations are required to confirm these findings and assess their generalizability.

In conclusion, using a prospective, nationwide, multicenter, OHCA registry in Japan, we focused on sex-related differences in patients with OHCA with shockable rhythm at the scene. After adjusting for prognostic factors, we found no difference in the survival rates or hospital interventions between men and women.

## Data Availability

The original contributions presented in the study are included in the article/supplementary materials, further inquiries can be directed to the corresponding author.
